# Self-directed learning in post-graduate medical education: Self-judgement and supervisor judgement of competence development in Austrian nine-month basic training

**DOI:** 10.3205/zma001697

**Published:** 2024-09-16

**Authors:** Gudrun Khünl-Brady-Ertl, Reinhard Oeser, Barbara Seemann-Hlawati, Katja Varga, Michaela Wagner-Menghin

**Affiliations:** 1 Vienna Healthcare Group, General Directorate, Human Resources Development and Training Department, Vienna, Austria; 2 Fa. Oeser GmbH, Vienna, Austria; 3 Vienna Healthcare Group, Klinik Landstraße, Medical Directorate, Vienna, Austria; 4 Medical University of Vienna, Clinical Division for Social Psychiatry, Department of Psychiatry and Psychotherapy, Vienna, Austria

**Keywords:** workplace-based learning, self-directed learning, postgraduate medical education and training, basic training, logbook, reflection, self-judgement, self-assessment

## Abstract

**Background:**

Self-directed learning in the workplace should lead to the mastery of predefined learning objectives, with subjective competence judgements steering learning and promoting acceptance of feedback. Rotations should support self-directed learning in basic training by allowing junior physicians (JPs) to apply basic clinical competencies in various internal medicine and surgical departments.

**Aim:**

The study hypothesises that rotations support self-directed learning, as measured by self-judgements and supervisor judgements. Additionally, it describes JPs’ willingness to reflect on their learning needs at the end of their basic training.

**Methods:**

This longitudinal study comprises 147 pseudonymised logbooks completed by JPs from three Vienna healthcare group (WIGEV) clinics. The logbook accompanies JPs’ training, requiring them and their supervising specialist physicians to rate their training goal completion (10-level % scale) in training months 2, 3, 5, 6, 8 and 9. In addition, in months 3, 6 and 9, the JPs document the level of competence (knowledge, experience and proficiency) they feel they have achieved for each learning objective specified by the Austrian medical association (ÖÄK).

**Results:**

The self-judged level of training goal completion demonstrates a multi-peaked distribution with an increasing trend; the supervisors’ judgement of JPs’ level of training goal completion is almost parallel. The share of learning objectives where the required level of competence is seen as not yet mastered decreases throughout the training. In the 9^th^ month of training, approximately ¼ of the JPs indicated a need to learn in ≥10% of the learning objectives, independent of the training clinic.

**Conclusions:**

After switching departments, JPs downgraded their rating of training goal completion. Rotation supports realistic self-judgement, as competencies must be applied and reassessed in a new context. Most JPs consider the required level of competence per learning objective to be mastered at the end of basic training, yet they remain prepared to reflect critically on their learning needs.

## Zusammenfassung

**Hintergrund:**

Bei selbständiger Erarbeitung vorgegebener Ausbildungsziele am Arbeitsplatz steuert die subjektive Kompetenzeinschätzung das Lernen und das Annehmen von Feedback. In der Basisausbildung sollen Rotationen das selbstgesteuerte Lernen unterstützen, indem klinische Basiskompetenzen in verschiedenen internistischen und chirurgischen Abteilungen einzusetzen sind.

**Ziel:**

Die Studie untersucht anhand von Selbst- und Fremdeinschätzung die Hypothese, dass Rotationen selbstgesteuertes Lernen unterstützen. Zusätzlich wird die Bereitschaft der Turnusärzt*innen (TÄ) zur Reflexion des Lernbedarfs am Ende der Ausbildung pro Ausbildungsklinik beschrieben.

**Methode:**

Die Längsschnittstudie umfasst 147 pseudonymisierte Logbücher von TÄ aus drei Kliniken des Wiener Gesundheitsverbunds (WIGEV). Das ausbildungsbegleitende Logbuch fordert TÄ und supervidierende Fachärzt*innen (FÄ) in den Ausbildungsmonaten 2, 3, 5, 6, 8 und 9 auf den Grad der Erfüllung des Ausbildungsziels (10-stufige %-Skala) einzuschätzen. Zusätzlich dokumentieren in den Monaten 3, 6, und 9 die TÄ welches Kompetenzniveau (Kenntnis, Erfahrung, Fertigkeit) sie für die von der österreichischen Ärztekammer (ÖÄK) vorgegebenen Ausbildungsinhalte erreicht sehen.

**Ergebnisse:**

Der Grad der Erfüllung des Ausbildungsziels, selbst eingeschätzt durch die TÄ, verläuft mehrgipflig mit steigendem Trend, die Einschätzung der FÄ verläuft annähernd parallel. Der Anteil der Ausbildungsinhalte, in denen das vorgegebene Kompetenzniveau als noch nicht erreicht gesehen wird, sinkt im Verlauf der Ausbildung. Im 9. Ausbildungsmonat sehen ca. ¼ der TÄ in ≥10% der Ausbildungsinhalte noch Lernbedarf, unabhängig von der Ausbildungsklinik.

**Schlussfolgerungen:**

TÄ revidieren nach Abteilungswechsel die Einschätzung des Erfüllungsgrads nach unten. Die Rotation unterstützt die realistische Selbsteinschätzung, indem Kompetenzen in neuem Kontext eingesetzt und neu bewertet werden müssen. Mehrheitlich sehen TÄ das Zielniveau pro Ausbildungsinhalt am Ende der Basisausbildung erreicht, sind jedoch bereit zu kritischer Reflexion des Lernbedarfs.

## Introduction

Self-directed learning is essential for lifelong learning in the medical profession [1], [2]. In workplace-based training, trainees achieve the specified competence level for the respective training step for each learning objective (e.g. Austrian medical training regulations, current version) [3], by applying their medical knowledge under supervision. However, as medical care takes centre stage in the workplace, learning in this environment places particular demands on the ability to self-direct one’s learning [4]. The starting point for self-directed learning is setting goals, which must then be pursued in a planned and persistent manner. Reflection, self-judgement and self-assessment significantly contribute to this because their results inform planning and regulating behaviour [2], [5]. A review of self-directed, supervised learning in the clinic concludes that using learning goals, documenting one’s learning process, setting personal sub-goals as well as planning and persistently pursuing these goals are effective. However, empirical results are still lacking on self-assessment and self-judgement as essential steps in self-directed learning [4].

Studies on self-assessment among trainees in post-graduate training initially highlight that those rated by experts as above average are very self-critical of their performance after completing a clinical exercise, such as delivering bad news and simulation with standardised patients, while those rated as below average tend to overestimate themselves [6], [7]. Combined with external feedback, however, self-judgement is crucial in competence development [7]. The correspondence between self-judgement and supervisor judgement also increases when learners are familiar with the possible range of performance in the exercise [6] and with the task [8], [9]. Furthermore, the ability to self-judge accurately depends on age and study length and increases throughout the programme [9]. 

Two cross-sectional studies – one from Austria on 6^th^-year medical students and physicians in basic training, general practice training and speciality training [10], and one from Germany on physicians undergoing specialist training in general medicine [11] – examined the self-assessed achievement of learning objectives in the curriculum. They concluded from the comparison of sub-groups with different clinical experience or levels of training that more experienced trainees feel more confident in several competencies than less experienced trainees. In a Dutch longitudinal study, the subjectively assessed competence of primary care physician trainees increased from “moderate-adequate” to “adequate-good” throughout the three-year training period [12].

**Figure 1:**
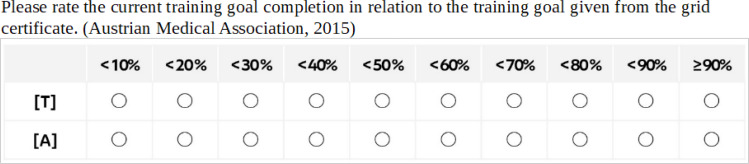
Percentage scale for global rating of training goal completion across all learning objectives in 10 intervals, judged by [T] JPs and [A] supervising SPs. Ratings are performed during mid-term and final evaluations within the department.

**Figure 2:**
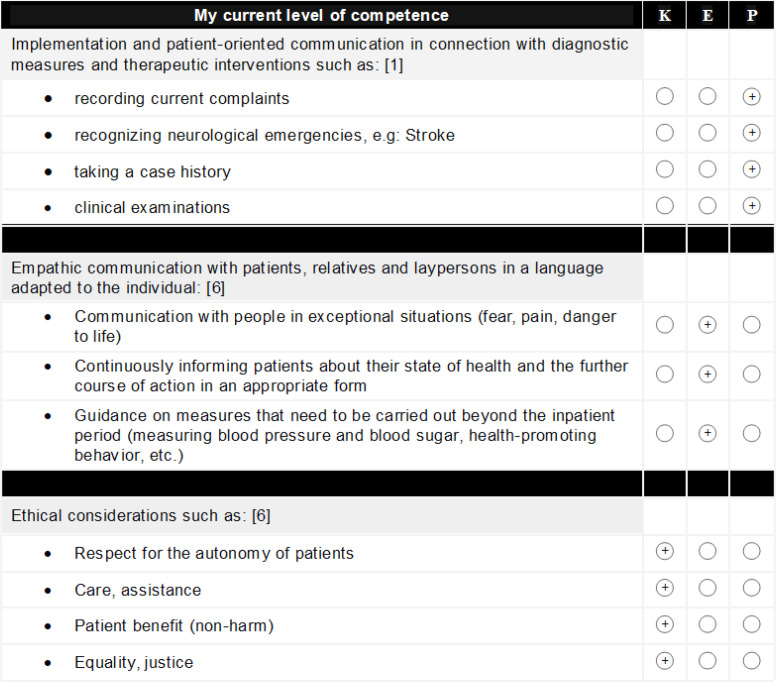
Examples from the catalogue of learning objectives in the basic training logbook used for the subjective evaluation of competence levels achieved for each learning objective. One example is provided for each competence group (K, E and P). The complete catalogue is included in the logbook. Identical lists are used for self-judgement at 3, 6 and 9 months.

Based on the 2015 medical training regulations (ÄAO), post-graduate medical training begins with basic training, which is accompanied by a logbook [3], [13]. In the logbook of the Vienna healthcare group (WIGEV), the learning objectives specified by the Austrian medical association (ÖÄK) are grouped following the three competence levels to be achieved: “knowledge”, “experience” and “proficiency” (K, E and P, original: Kenntnis, Erfahrung, Fertigkeit, K, E and F) [14], [15], [16], [17]. These objectives are accompanied by self-judgement and supervisor judgement scales (see figure 1 (Fig. 1)) as well as structured (see figure 2 (Fig. 2)) and free reflection tasks. The WIGEV junior physicians (JPs) rotate through three departments for three months each. Predefined training activities are applied in a structured and documented manner in each department. On the one hand, clinical tasks with immediate reflection and feedback are performed. On the other hand, a mid-term and final evaluation is conducted in each department, for which JPs prepare themselves using the rating scales and the structured reflection tasks to collect their supervising doctor's judgement and feedback [14], [15], [16], [17].

This study analyses the effect of rotation on competence development based on self-judgement and supervisor judgement. Based on a Dutch follow-up study, an increase in the self-judged level of completion of the training objectives during training is assumed [12]. Furthermore, self-judged competencies and the judgement by supervising specialists are assumed to be similar (hypotheses 1 and 2).

Based on the results of cross-sectional studies [10], [11] showing an increase in competence assessment with greater clinical experience, the study examines hypothesis 3: JPs perceive an increase in competence for most learning objectives as their experience grows throughout training. 

The study also describes the willingness of JPs to reflect on the achievement of learning objectives (hypotheses 4&5). Thus, it examines whether JPs subjectively consider the training goal to have been achieved at the end of basic training or still identify a need for further learning. The study will derive measures to improve the quality of training and strategies to strengthen the perception of the logbook as a helpful tool for self-directed learning in the workplace.

## Method

### Sample

This descriptive field study with longitudinal design analysed logbooks (n=147) submitted by JPs in basic training who started their training in 2016, 2017 and 2018 (n=190) in three WIGEV clinics: Klinik Landstraße (KLA), Klinik Donaustadt (KDO) and Klinik Ottakring (KOR). In those hospitals, the clinic's education and continuing education officer supported the survey throughout the period. Logbooks in which no training objective was completed in the 3^rd^, 6^th^ or 9^th^ month were excluded.

### Setting and procedure 

Basic training aims for JPs to master all learning objectives at the specified competence level. The WIGEV training logbook groups the ÖÄK's learning objectives into three competence levels (K, E and P) and combines these with two rating scales (see figure 1 (Fig. 1) and figure 2 (Fig. 2)). During the introduction, the clinic’s education and continuing education officers provide the “LOGBUCH Basisausbildung”, a logbook for basic training, directly to the JP, explain its use and the importance of continuous documentation and collect it for evaluation at the end as part of the final review of training. The logbook remains with the JPs for the entire training period and is maintained independently. One component of the mid-term evaluation (in months 2, 5 and 8) and the final evaluation (in months 3, 6 and 9) at the department is the global rating of training goal completion by the JPs and supervising specialist physicians (SPs). The JP performs additional reflection on the level of competence mastered for each learning objective towards the end of the 3^rd^, 6^th^ and 9^th^ months of training using the same rating scale (extract in figure 2 (Fig. 2)).

In the WIGEV basic training logbook, JPs document the results of structured training activities, such as onboarding and offboarding, mid-term and final evaluations, departmental rotations and clinical tasks, using free text questions. The results of these additional activities are not the subject of this study. 

### Measures

#### Level of training goal completion (LC)

This judgement is made by JPs and SPs using a 10-point scale (see figure 1 (Fig. 1)). The mean values (MW) for the variables “level of training goal completion” (JP and SP) are calculated using the respective class MW (calculation rule in the attachment 1 , table 1A) and expressed as percentages, with a maximum value of 95%. The variables are labelled based on the following pattern: *LC–JP (x)*: Global level of training goal completion in percentage based on self-judgement by JPs at the time point (x) or *LC–SP (x)*: Global level of training goal completion in percentage based on the judgement of supervising SPs at the time point (x).

#### Percentage mastery of learning objectives (MLO)

According to ÖÄK’s ÄAO 2015, the competence level (K, E and P) to be mastered is defined for 81 learning objectives [13]. The level of competence mastered is reflected upon using the structured reflection task in the logbook by JPs. Figure 2 (Fig. 2) shows examples from the learning objectives catalogue. The required competence level of “K” has to be mastered for 24 learning objectives and “E” and “P” for 20 and 37 objectives, respectively. The aggregation of information necessary to quantitatively test hypotheses (3, 4 and 5) was based on these three competence groups.

To calculate the “average mastery of learning objectives”, the competence level achieved based on self-judgement is related to the target competence level. Therefore, the 10-point rating scale is transformed into percentage values (scoring rule in the attachment 1 , table 2A). Each person's average mastery of learning objectives is calculated for each of the three competence groups at each measurement point. The variables are labelled based on the following pattern: *AMLO-K (x)*: Average mastery of learning objectives in percentage for objectives with the target level “K” at the time point (x). Furthermore, the value “AMLO-Total(x)” is calculated as the average mastery of learning objectives in percentage for all learning objectives at the time point (x). A value of 100% is achieved if the defined target level is achieved for all learning objectives in a group. The further the value deviates from 100%, the more learning objectives are not yet mastered at the target level.

#### Learning needs at the end of basic training

Mastering >90% of the learning objectives in each competence group at the end of training (9^th^ month) was a criterion for the “training goal met” in the study. A “learning need” arises at the end of basic training when ≤90% of the learning objectives have been mastered in one or more of the three competence groups (learning need ≥10%).

### Statistical evaluations and analysis

**Hypotheses 1 and 2:** To investigate the hypothesis of an increase in the subjectively perceived level of training goal completion or the level of completion as perceived by the supervising SPs, descriptive statistics (MW, standard deviation [SD], median and 2^nd^ and 3^rd^ quartile) are calculated for the two times six variables *LC–JP or LC–SP*. A box plot is drawn, and the Spearman rank correlation is calculated.

**Hypothesis 3:** To investigate the hypothesis of an increase in subjectively perceived mastery of learning objectives throughout training, descriptive statistics (MW, SD, median and quartile) are calculated for the nine variables *AMLO-K/E/P (x)*. Box plots are created for each competence group and the Spearman rank correlation is calculated using the three characteristic values AMLO-Total (x). 

**Hypotheses 4 and 5:** To test these hypotheses, the frequencies for the variable “training goal met” versus “learning need” at the end of basic training are presented in a table 1 (Tab. 1) (total and divided by clinic).

Microsoft Access 365^®^ is used for data management, and Microsoft Excel 365^® ^and NCSS 2022 statistical software are used for data analysis. The data processing is pseudonymised; the evaluation results in this work do not allow any conclusions to be drawn about specific individuals.

## Results

### Increase in the level of training goal completion in the global self-judgement and supervisor judgement throughout the training (hypotheses 1 and 2)

**Figure 3:**
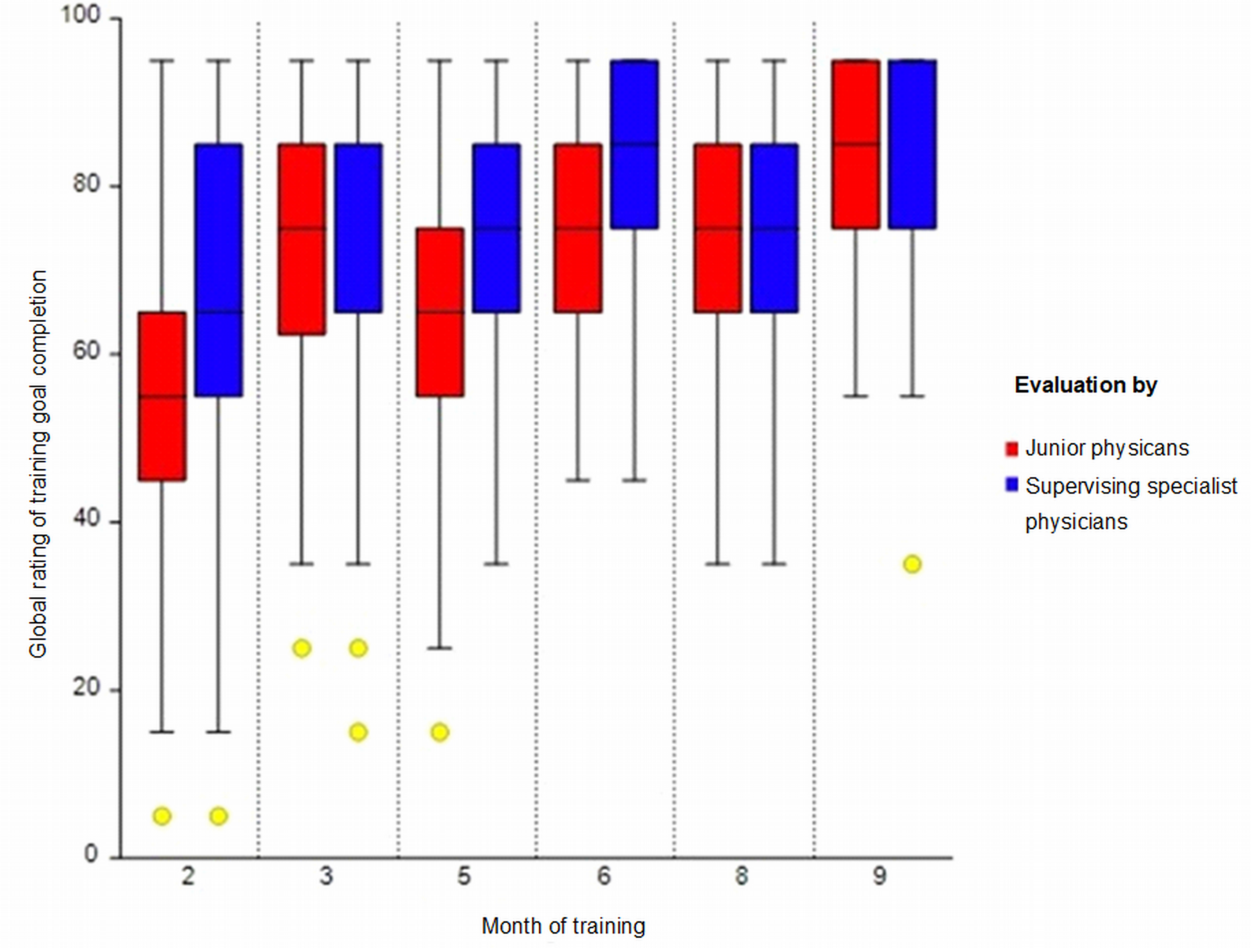
Comparison of training goal completion: Self-judgement by JPs versus supervisor judgement by supervising specialists. Each boxplot shows the average ratings of self-judgement (red) and supervisor judgement (blue). The box represents the 25^th^, 50^th^ and 75^th^ percentiles of self-judgement. Dots indicate outliers. The figure illustrates that JPs rate themselves more critically than their supervisors rate them. Note: For an interpretation of the box plots, see attachment 1, table 4A

The descriptive results on the level of training goal completion, self-judged by the JPs and the supervising SPs at the six measurement points (in months 2, 3, 5, 6, 8 and 9), as part of the mid-term and final evaluations at the training departments, can be seen in the box plot in figure 3 (Fig. 3) (MW and SD in the attachment 1 , table 3A). Based on the supervisors’ judgement, the level of training goal completion does not increase monotonically. Instead, multi-peaked development can be observed (see figure 3 (Fig. 3), red boxes). After a change of department (after the 3^rd^ and 6^th^ months), the JPs downgrade their judgement of the level of completion. Between measurement points 3, 6 and 9, the expected increase in training goal completion can be observed along with a reduction in the dispersion and a lower number of outliers.

The judgement of the supervising SPs was conducted by different supervising SPs in various departments. Interestingly, the judgement in the 5^th^ and 8^th^ months (after a department change) resulted in the same median, although with fewer downward outliers in the 8^th^ month. At the end of the training period in the second department (6^th^ month), the average judgement of the JPs was the same as at the end of the training period in the third department (9^th^ month) but with less variation. The MW of the supervisors’ judgements displayed less variance than those of the JPs’ self-judgements. However, they were similar (see figure 3 (Fig. 3), blue boxes).

The Spearman rank correlation between the self-judged level of training goal completion (LC–JP) and training time (6 measurement points) is r=0.35 (df=880, p<0.001, n=882, r^2^=0.12, weak effect). Therefore, the hypothesis of independence between measurement time points and self-judgement (H0) can be rejected. The corresponding results for the global judgement of the level of training goal completion by the supervising SPs (LC–SP) and training time (6 measurement points) are r=0.25 (df=880, p<0.001, n=882, r^2^=0.06, very weak effect). These results also support the rejection of the hypothesis of independence between measurement points and judgement.

### Subjectively perceived increase in competence during training (hypothesis 3)

**Figure 4:**
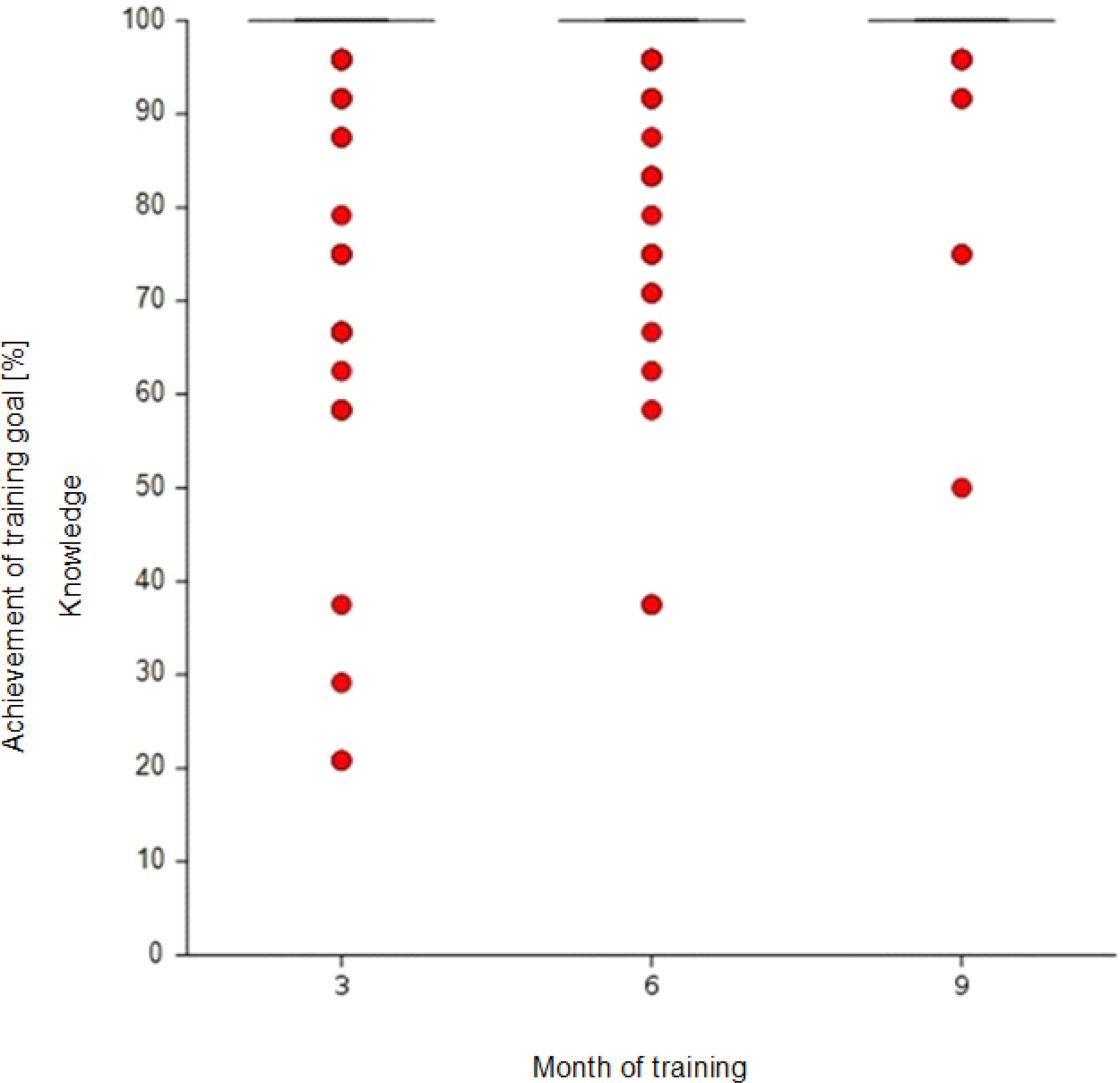
Competence development throughout basic training: Self-judgement on learning objectives with a target level of “K” at the end of the 3^rd^, 6^th^ and 9^th^ months. In the 3^rd^ month, the median, 75^th^ percentile and 25^th^ percentile overlap at 100%. The number of outliers decreases over time. Note: For an interpretation of box plots, see attachment 1, table 4A

**Figure 5:**
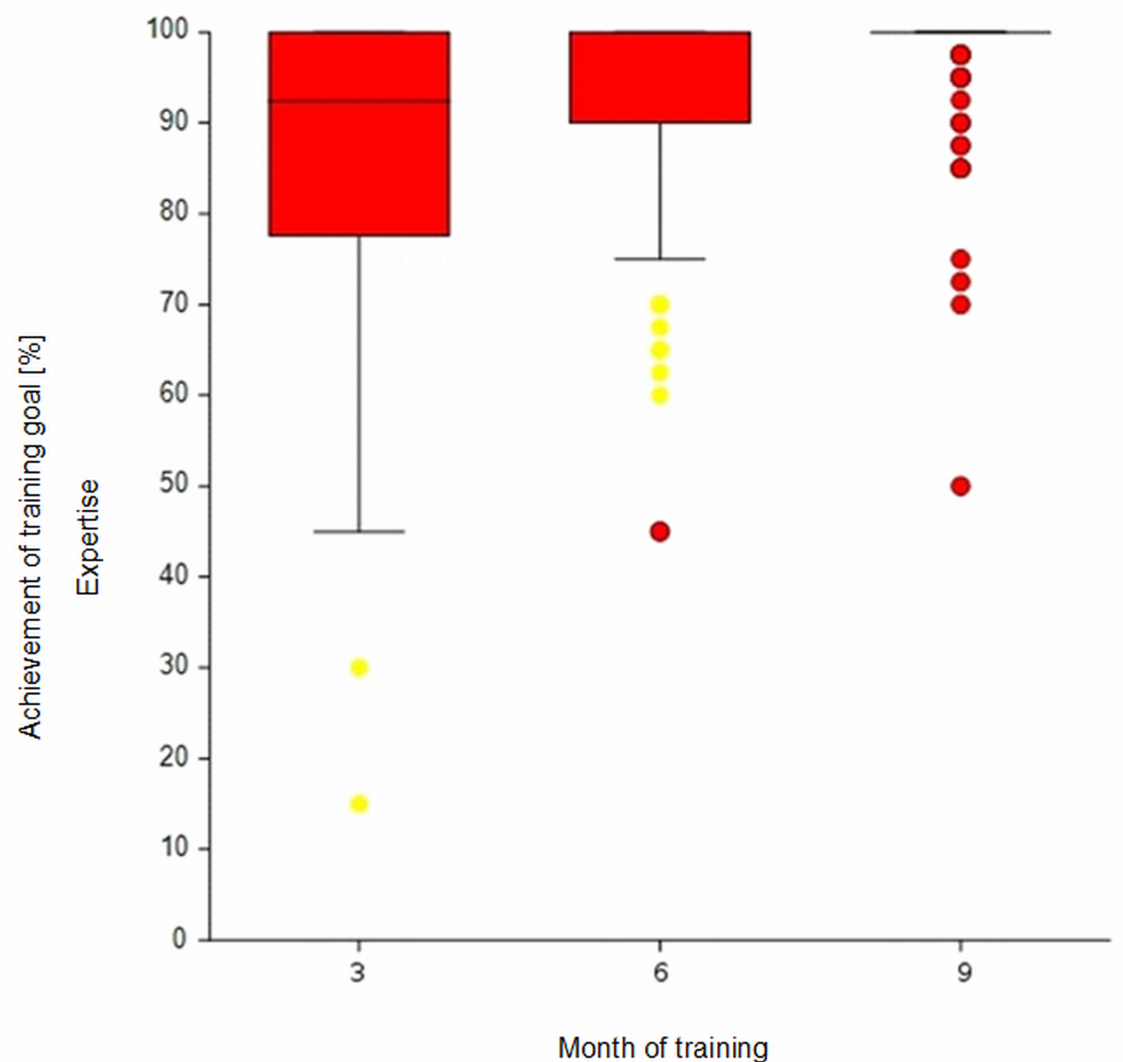
Competence development throughout basic training: Self-judgement on learning objectives with a target level of “E” at the end of months 3, 6 and 9. Notably, in months 6 and 9, the median and 75^th^ percentile are at the 100% mark. In month 9, the 25^th^ percentile also coincides with the median. Note: For an interpretation of box plots, see attachment 1, table 4A

**Figure 6:**
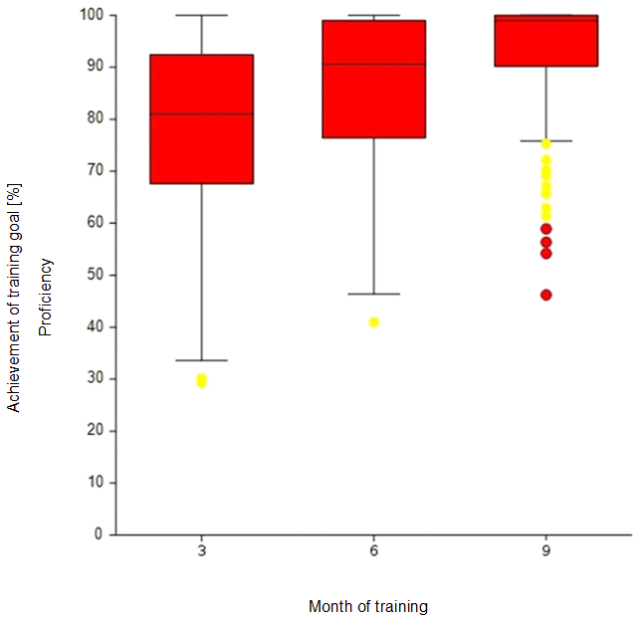
Competence development throughout basic training: Self-judgement on learning objectives with a target level of “P” at the end of the 3^rd^, 6^th^ and 9^th^ month. Note: For interpretation of box plots, see attachment 1, table 4A

The descriptive results of the self-judged level of competence mastery per competence group (K, E and P) after 3, 6 and 9 months of basic training are shown in the box plots in figure 4 (Fig. 4), figure 5 (Fig. 5) and figure 6 (Fig. 6) (MW and SD in the attachment 1 , table 5A). Subjectively perceived competence increase can be observed for all three competence groups. After three months, JPs were perceived to have mastered 94% of the learning objectives in the K group. This value increased to 99% after nine months. The dispersion decreased across the three measurement points, and fewer outliers were observed. The same effect could also be seen for the E (87%-98%) and P (79%-93%) competence groups.

The Spearman rank correlation between the self-judged level of competence achieved (total score comprising all learning objectives) and training time (3 measurement points) is r=0.39 (df=439, p<0.001, n=441, r^2^=0.15, weak effect). Therefore, the hypothesis of independence between measurement points and self-judgement (H0) can be rejected.

### At the end of basic training, most JPs considered the training goal achieved, and any perceived learning needs did not differ between the three clinics (hypotheses 4 and 5)

After nine months of basic training, most JPs (74.15%) considered the learning goal achieved. However, 25.85% of the JPs still perceived a need to learn over 10% of the learning objectives. At 23.08%, the least JPs in KDO reported a need to learn, while the most JPs in KLA reported a need to learn at 29.17%. The difference of 6.09 percentage points is within the previously defined range of a maximum 10% difference (see table 1 (Tab. 1)), implying that the quality of the training provided to the JPs at the three clinics is comparable.

The MW of the subjectively perceived competence per competence group (K, E and P) or total at the end of training (measurement point 9, *AMLO-K/E/P* [9]) for each clinic or across all clinics allows for a more detailed exploration of the JPs’ learning needs. The MW showed that the greatest need for learning is seen in the P group. The variance in subjectively perceived competence is also more pronounced for this learning objectives group than for the K and E groups.

## Discussion

In the first stage of the Austrian post-graduate medical training, the basic training, JPs participated in patient care under supervision, performing tasks tailored to their level of training. During the rotation through various departments, all JPs must acquire basic clinical competencies as a requirement for subsequent specialist training. Increasing responsibility in the workplace requires regular subjective judgement of competencies to promote self-directed learning and the acceptance of feedback [7]. This longitudinal study is the first to use self-judgements to investigate the effect of the 3×3-month rotation format in WIGEV on developing basic clinical competencies and the willingness of JPs to reflect on their learning. Two self-rating scales from the “LOGBUCH Basic Training” developed at WIGEV were used for this purpose [14], [15], [16], [17].

The global judgement of training goal completion, assessed at six different points, showed a downward correction in self-judgements in months 5 and 8 after department changes in months 3 and 6. The results indicate that the self-judgements of the JPs vary depending on external factors, highlighting successful self-directed learning [8], [9]. Different challenges in the various departments require an adjustment in the global rating of one’s level of training goal completion, which can be interpreted as an effect of rotation.

When interpreting the supervising physicians’ global judgements, it must be considered that, due to the rotations, JPs are evaluated by at least three SPs. The judgement is part of the mid-term and final evaluations at each department. Notably, WIGEV SPs are experienced in evaluating JPs’ current competence level within the ÖÄK learning objectives. This is reflected in the lower variance of the MW of the JPs’ competence as judged by the SPs. Supervisor judgements, generally very close to JPs’ judgements, were documented as part of feedback in the logbook to promote accurate self-judgement [7].

The third hypothesis that JPs experience an increase in their competence during basic training was confirmed by the self-judged level of competence achieved per learning objectives after 3, 6 and 9 months. The average achievement of learning objectives per competence group (K, E and P) increased throughout the training. After three months, the average mastery for all three competence areas was 86%, rising to 96% after nine months. These results aligned with expectations that workplace-based training increases competencies.

Hypotheses 4 and 5 address subjective competence and perceived learning needs at the end of basic training and the differences between the clinics. Just under ¾ of the JPs stated they subjectively achieved >90% of the learning objectives, indicating little or no need to learn further. However, about ¼ still perceived a need to learn in ≥10% of the learning objectives. This demonstrates the JPs’ willingness to self-judge themselves and openly state their learning needs [11]. The results were similar in the three clinics, highlighting a comparable quality of basic training.

Overall, the study shows that JPs experience a subjective increase in their competencies during basic training and are prepared to make realistic self-judgements. Based on the results, external factors modify JPs’ self-judgements during basic training. Therefore, the logbook is an instrument to support self-directed learning and ensure training quality. Redundancy between the “clinical practical year” in the 6th year of study and basic training cannot be deduced from this study, even if the self-judgements indicate high levels of mastery after three months.

### Limitations

This field study evaluated the subjective development of competencies, willingness to self-judge during basic training and rotation’s effect on self-judgements for the first time. However, the underlying individual mechanisms and the influence of departmental or system-level measures on competence development were not examined. One limitation of the self-judging method is that JPs may not have documented subjective learning needs for fear of negative consequences. To minimise this risk, procedural instructions for using the logbook include precautions to alleviate such fears. The logbook is completed independently by the JPs, and feedback from the supervising SPs, such as the global judgement of the level of completion of learning objectives using a 10-point scale, is entered in the logbook by the JPs themselves during mid-term and final evaluations at the departments.

Self-judgement of competencies before or at the beginning of basic training is needed for a complete presentation of competency development. Similarly, it remains unclear which learning objectives JPs perceive a need to learn and whether a correlation exists between these perceived learning needs, departments and rotation patterns. This should be the subject of further research.

## Conclusions

Open and routine self-judgement of competencies is essential for identifying competency gaps at the individual level. The logbook-accompanied basic training with a structured 3×3-month cycle at WIGEV supports the development of realistic self-judgement using competencies in different contexts. The WIGEV's “LOGBUCH Basisausbildung” implements legal requirements and structures departmental stays through didactic concepts for conveying training objectives. It also triggers regular reflection on achieving learning objectives through structured self-judgement tasks. Using these tasks, the study illustrated the subjective development of competencies throughout basic training. In addition, the results from evaluating these tasks are informative for WIGEV, Austria’s largest provider of post-graduate medical education, to further develop the quality of basic training. The basic training logbook, which allows for documenting results from structured training activities, could be modified as a valuable tool for self-directed learning and quality assurance in other training contexts and thus initiate the improvement of the multi-phase training existing in Austria since 2015. 

## Legal basis of Austrian medical training

- Physicians Act 1998 (ÄrzteG 1998). Query on 22 October 2022. Federal Law Gazette I No. 169/1998 [https://www.ris.bka.gv.at/Dokumente/BgblPdf/1998_169_1/1998_169_1.pdf]
- FAQs on doctors’ training regulations, status: November 2019. Query on 22 October 2022. [https://www.aerztekammer.at/aeao-2015]
- ÖÄK_Basisausbildung training book. RIS. Query on 10 October 2021. [https://www.aerztekammer.at/documents/261766/88693/%C3%96%C3%84K_Basisausbildung+Ausbildungsbuch_20151111.pdf/c8fe3753-718f-86a6-8e4b-91c9a57bc3fd]
- RZ_Basisausbildung V1.0. RIS [https://www.aerztekammer.at/basisausbildung]
- Ordinance of the Federal Minister of Health on the Training of General Practitioners and Specialists (General Practitioner Training Regulations 2015 - ÄAO 2015). Query on 22 October 2022. Federal Law Gazette II No. 147/2015 [https://www.ris.bka.gv.at/eli/bgbl/II/2015/147], [https://www.ris.bka.gv.at/GeltendeFassung.wxe?Abfrage=Bundesnormen&Gesetzesnummer=20009186]
- Regulation of the Austrian Medical Chamber on knowledge, experience and skills in the training of general practitioners and specialists as well as on the design and form of the grid certificates, examination certificates and training books (KEF and RZ-V 2015). Query on 22 February 2022, [https://www.aerztekammer.at/documents/261766/417698/KEF_RZ+VO+2015+3_ueWB_Nov+2019-12-13+Beschl+140.+VV_konsolidiert+samt+allen+Anlagen.pdf/a999fc10-7fad-ed7e-9aee-b58c87d31eb4?t=1576754313029]

## Authors’ ORCIDs

- Gudrun Khünl-Brady-Ertl: [0009-0002-1639-9215]
- Reinhard Oeser: [0009-0000-8343-6368]
- Michaela Wagner-Menghin: [0000-0003-1645-7577]

## Competing interests

The authors declare that they have no competing interests. 

## Supplementary Material

Supplementary tables

## Figures and Tables

**Table 1:**
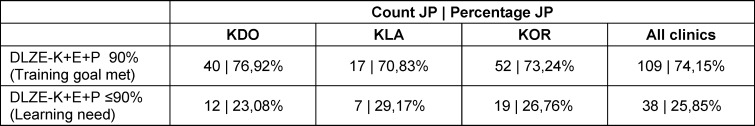
Frequencies (%) of JPs who, based on self-judgement, have mastered over 90% of the learning objectives at the specified competence level and thus achieved the training goal or have mastered below 90%, indicating a learning need for more than 10%of the learning objectives. Values are provided for each clinic (KDO, KLA, KOR) and overall.
