# Supplementary material for: Self-directed learning in post-graduate medical education: Self-judgement and supervisor judgement of competence development in Austrian nine-month basic training
Source: GMS J Med Educ. 2024 Sep 16;41(4):Doc42. doi: 10.3205/zma001697 (PMC11474653; doi:10.3205/zma001697)
Supplement: Supplementary tables [file JME-41-42-s-001.pdf]

Attachment 1: Supplementary tables

Table 1A: Calculation rule for transforming the ordinal percentage scale into a class mean value for the global judgement of training goal completion

**Line missing value 0%**	
< 10%→ 5%	< 60%→ 55%
< 20%→ 15%	< 70%→ 65%
< 30%→ 25%	< 80%→ 75%
< 40%→ 35%	< 90%→ 85%
< 50%→ 45%	≥ 90%→ 95%

Note: The percentage scale for global judgement is divided into 10 intervals. For this study, the class average value is used. For instance, a range of <=50% to <60% is represented as 55%. Therefore, the maximum achievable value is capped at 95%.

Table 2A: Calculation rule for transforming the ordinal scale used for judging competence (K, E, P) in training content based on ÖÄK training regulations

	**Achieved level**		
**Target level**	**K (1)**	**E (2)**	**P (3)**
K (1)	100%	100%	100%
E (2)	50%	100%	100%
P (3)	24%	60%	100%

Note: By capping the target level for K and E at 100%, we ensure that 'over-achievement' in one area does not compensate for unmet learning objectives in other competence levels. Training content for which no competence level is assigned is evaluated with a target achievement of 0%, indicating a potential lack of knowledge in that area. For instance, if a trainee self-assesses as reaching the target level E but with only K for specific training content, this implies that the target has been incompletely achieved. Following the calculation rule, such content was assessed as 50% achieved. If the trainee self-assesses at level [P] proficiency, they have surpassed the target, but the achievement is capped at 100%.

Table 3A: Global ratings of training goal completion (LC) in percentage (%): Judgements by JP and SP

**Count = 147 MW (SD) [%]**	**Two months**	**Three months**	**Five months**	**Six months**	**Eight months**	**Nine months**
LC-JP %	55 (22)	66 (24)	62 (21)	72 (24)	66 (25)	80 (22)
LC-SP %	61 (62)	64 (33)	66 (26)	71 (30)	69 (27)	76 (30)

Attachment 1 to Khünl-Brady-Ertl G, Oeser R, Seemann-Hlawati B, Varga K, Wagner-Menghin M. *Self-directed learning in post-graduate medical education: Self-judgement and supervisor judgement of competence development in Austrian nine-month basic training.* GMS J Med Educ. 2024;41(4):Doc42. DOI: 10.3205/zma001697

Table 4A: Interpretation of box plots

- The box represents the interquartile range (IQR), which covers the central 50% of the data.
- The line inside the box indicates the median value.
- The whiskers extend from the edges of the box to the minimum and maximum data points within 1.5 times the IQR.
- Mild outliers, marked in yellow, lie between the end of the whiskers and the start of the strong outliers.
- Strong outliers, marked in red, fall beyond three times the IQR from the edges of the box.
- The IQR is the difference between the 1st quartile (Q1) and the 3rd quartile (Q3) and corresponds to the length of the box.

Table 5A: Mean and SD of "Average Mastery of Learning Objectives" (AMLO) overall and by competence group across three survey dates

	**MV (SD) [%]**		
	**Three months**	**Six months**	**Nine months**
AMLO-K	94 (15)	97 (10)	99 (05)
AMLO-E	87 (16)	93 (12)	98 (06)
AMLO-P	79 (18)	86 (15)	93 (11)
AMLO total	86 (14)	91 (10)	96 (07)

Note: K=Knowledge, E=Experience, P=Proficiency, Total=All Training Content (n=147 logbooks)

Attachment 1 to Khünl-Brady-Ertl G, Oeser R, Seemann-Hlawati B, Varga K, Wagner-Menghin M. *Self-directed learning in post-graduate medical education: Self-judgement and supervisor judgement of competence development in Austrian nine-month basic training.* GMS J Med Educ. 2024;41(4):Doc42. DOI: 10.3205/zma001697
